# Digital Light Processing (DLP) 3D Printing of Caprolactone Copolymers with Tailored Properties through Crystallinity

**DOI:** 10.1021/acsapm.4c01772

**Published:** 2024-09-16

**Authors:** Gianluca Bartolini Torres, Smiljana Stefanovic, Bo Li, Andreas Heise

**Affiliations:** † Department of Chemistry, RCSI University of Medicine and Health Sciences, Dublin, D02 YN77, Ireland; ‡ Science Foundation Ireland (SFI) Centre for Research in Medical Devices (CURAM), RCSI, Dublin, D02 YN77, Ireland; § AMBER, The SFI Advanced Materials and Bioengineering Research Centre, RCSI, Dublin, D02 YN77, Ireland

**Keywords:** polycaprolactone (PCL), 3D printing, copolymers, shape-memory, crystallinity, thiol−ene cross-linking

## Abstract

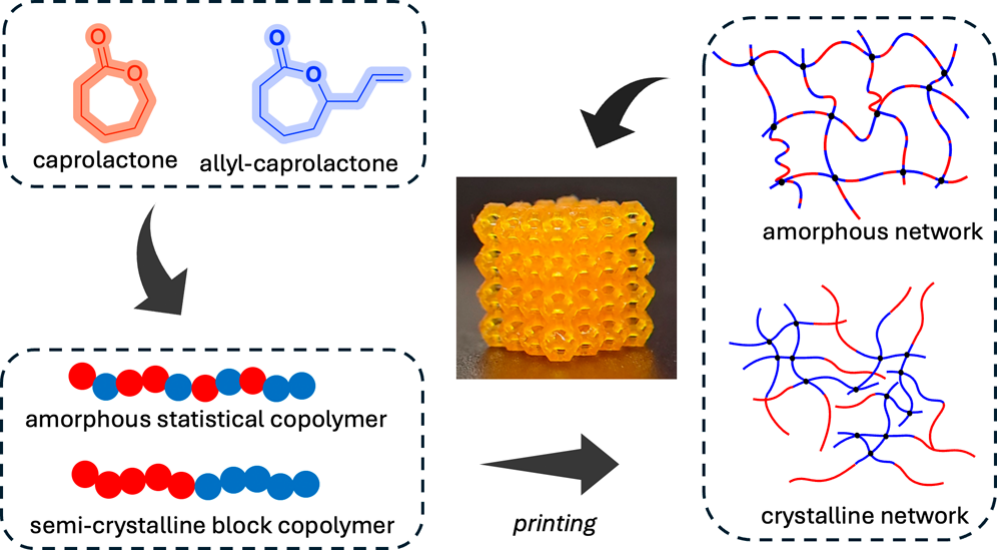

Digital light processing (DLP) 3D printing has shown great advantages such as high resolution in the fabrication of 3D objects toward a range of applications. Despite the rapid development of photocurable materials for DLP printing, tailoring properties to meet the specific demands for various applications remains challenging. Herein, we introduce copolymers of caprolactone and allyl caprolactone offering built-in functionality for thiol–ene photochemistry, thereby omitting the need for postfunctionalization. A crystalline block copolymer and an amorphous statistical copolymer were synthesized with the same comonomer composition and molecular weight. Thio–ene photocuring with a tetrafunctional thiol cross-linker was studied at different thiol to double-bond ratios for the copolymers and their blends. All formulations undergo rapid photocuring within several seconds of irradiation with slightly higher gel fractions observed for the statistical copolymer over the block copolymer under the same conditions, suggesting a somewhat higher cross-link density. Thermal properties of the networks were dependent on the presence of the semicrystalline block copolymer, where higher melting enthalpies were reached at higher block copolymer content. Similarly, crystallinity was found to be the main contributor to the mechanical properties. For a comparable composition, the modulus of a block copolymer network was found to be 31 times higher than that of the statistical copolymer network (27.7 vs 0.89 MPa). Intermediate moduli could be obtained by blending the two copolymers. DLP-printed scaffolds from these copolymers retained their thermal properties, therefore offering an efficient approach to tailoring mechanical properties, through crystallinity. Moreover, the printed scaffold displayed shape memory properties as the first example of poly(carprolactone) (PCL) copolymers in DLP printing. These materials are readily synthesized, offer fast and high-resolution 3D printing, and are degradable and cell compatible. They offer a straightforward approach to tailoring properties of PCL-based biomaterials and devices.

## Introduction

Digital light processing (DLP) 3D printing stands out among the available 3D printing technologies, as it facilitates printing of complex design structures with high resolution.^1−4^ DLP operates by exposing a photocurable resin to a series of images projected from a light source.^5^ Common DLP resins consist of commercially available photoreactive monomers such as (meth)acrylates and an epoxy/nucleophile.^6,7^ A barrier to broadening the scope of DLP lies in the limited availability of printable resins with adjustable properties.^8,9^ To meet the needs of advanced applications, the development of DLP resins with a wider property spectrum, including mechanical properties, degradability, recyclability, or biocompatibility, is critical.^10−12^ In that context, the development of photocurable resins from noncommercial feedstocks is of high interest.^13,14^ A promising recent development is the utilization of the growing pool of biomass-derived printable resins as renewable and environmentally resorbable feedstock.^15−18^ Examples include the incorporation of photoreactive groups into hyaluronic acid for tissue engineering applications^19^ or biomass materials such as phenolics and lignin.^20,21^ Notably, (meth)acrylation is usually the method of choice to introduce photoreactive groups and render a material DLP processable by light-induced radical polymerization. Despite the fast kinetics inherent to free radical polymerization for transforming resins into defined 3D structures, its main drawbacks include non-homogenous polymerization and the need for an excess of toxic molecules for postfunctionalization such as isocyanates and acryloyl chloride.^22^

Due to its biodegradability and biocompatibility, polycaprolactone (PCL) has evolved as a frequently used feedstock to fabricate biomaterials from 3D printing methods.^23,24^ However, most reported DLP printing processes of PCL require a postpolymerization end group (meth)acrylation, with the above-mentioned drawbacks. Manipulation of the mechanical properties of (meth)acrylated PCLs relies mainly on the variation of PCL molecular weight and cross-linking density.^25−27^ An alternative strategy for preparing photocurable resin is through thiol “click” chemistry.^28−31^ Compared to free radicals, the thiol–ene reaction allows the production of more homogeneous networks through a step-growth polymerization process with improved control over the cross-link density by adjusting the stoichiometry of the thiol and ene functional groups. As a consequence, the printed thermosets exhibit improved mechanical properties such as stiffness, flexibility, and toughness to meet the specific performance requirements for different applications.^32^ This concept has been successfully demonstrated for allyl-isocyanate functionalized PCL using a thiolate cross-linker.^33^ In this example, the elasticity of the DLP-printed thiol–ene network could be modified by changing the ratio of acrylate and thiol functionalities, allowing the fine-tuning of the network properties.

Here we introduce a copolymer comprising caprolactone (CL) and allyl caprolactone (ACL) allowing photoreactivity without the need of postfunctionalization.^34,35^ The developed copolymer resin system undergoes rapid photocuring through thiol–ene chemistry and is readily DLP printed. By altering the copolymer structure from an amorphous statistical to semicrystalline block copolymer, printed scaffolds with significantly different thermomechanical and degradation properties are obtained without changing the overall composition of the copolymers. Additionally, by the blending of statistical and block copolymers, intermediate material properties can be realized. The observed properties are determined by the overall crystallinity of the copolymers and their blends. The feasibility of this system is exemplified in the design of thermally responsive shape memory DLP-printed objects. The ability to significantly modulate the properties of the printed materials by copolymer chain structure without changing its overall monomer composition distinguishes our approach from commonly used (meth)acrylated PCL approaches.

## Results and Discussion

### Polymer Synthesis

**Figure 1:**
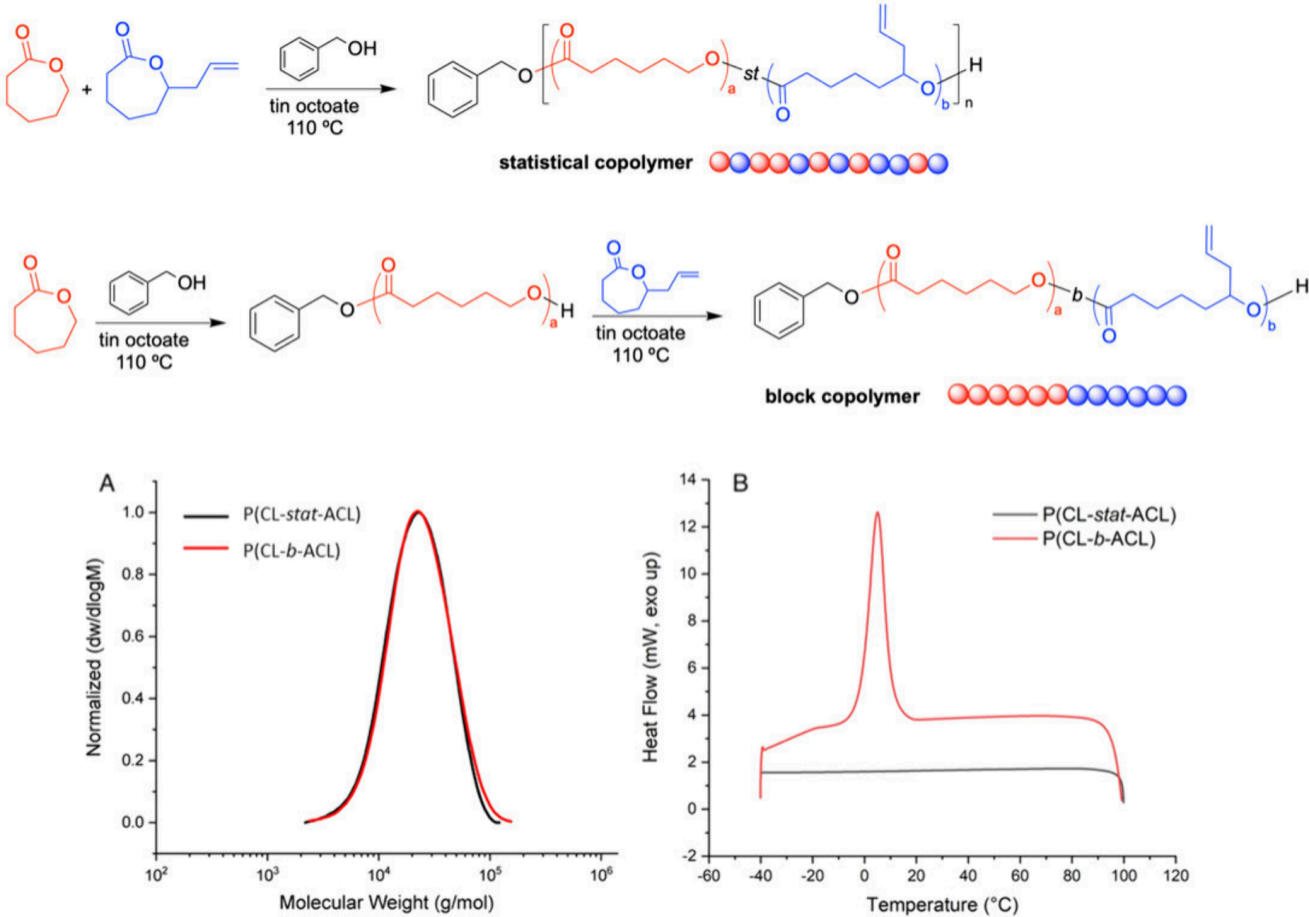
Synthesis of P(CL-*st*-ACL) and P(CL-*b*-ACL); (A) size exclusion chromatography (SEC) analysis of P(CL-*st*-ACL) and P(CL-*b*-ACL) (dRI detection, CHCl_3_, and PMMA standards). (B) Second cooling cycle of DSC of P(CL-*b*-ACL) and P(CL-*st*-ACL) copolymers.

Polymerizations were performed in bulk without prior drying of the reagents using tin(II)octoate as a catalyst and benzyl alcohol as initiator.^36^ Statistical copolymers of CL and ACL were obtained by the simultaneous polymerization of both monomers, while block copolymers were obtained by sequential monomer addition. Monomer conversion was monitored by ^1^H NMR spectroscopy by the disappearance of C*H*_2_O (δ = 4.15 ppm) and O=COC*H*O (δ = 4.25 ppm) ACL and CL signals, respectively (Figure 1A). A ratio of CL to ACL of 2:1 and a molecular weight of *M*_n_ 10,000 g mol^–1^ was targeted for both copolymers. The degrees of polymerization (*DP*) of CL (*DP* = 50) and ACL (*DP* = 25) were confirmed by end group analysis between the benzyl group (C=OOC*H*_2_Ar) (δ = 5.06 ppm), PCL (C*H*_2_OCO) (δ = 4.05 ppm), PACL (C*H*=CH_2_) (δ = 5.72 ppm), and PACL (CH=C*H*_2_) (δ = 5.18–5.01 ppm). The calculated *M*_n NMR_ of the statistical and block copolymer was 11,200 g mol^–1^ and 10,800 g mol^–1^, respectively, which are close to the targeted *M*_n_. DOSY NMR confirmed that no major water initiation occurred especially for the block copolymer synthesis, as only one polymer diffusion was observed from both block and statistical copolymers (Figure S2). Size exclusion chromatography (SEC) analysis displayed monomodal peaks (*Đ*_M_ = 1.7–1.8), suggesting no significant transesterification occurred in either copolymer (Figure 1A). Notably, both copolymers are indistinguishable by ^1^H NMR (Figure S3) and SEC analysis but differ in thermal properties based on their copolymer architecture, as evident from differential scanning calorimetry (DSC). DSC thermograms show a melting transition for the block copolymer due to the crystallization of the PCL block, which is absent in the amorphous statistical copolymer (Figure 1B).

### Resin Formulation, Photoreactivity, and Material Properties

**Figure 2:**
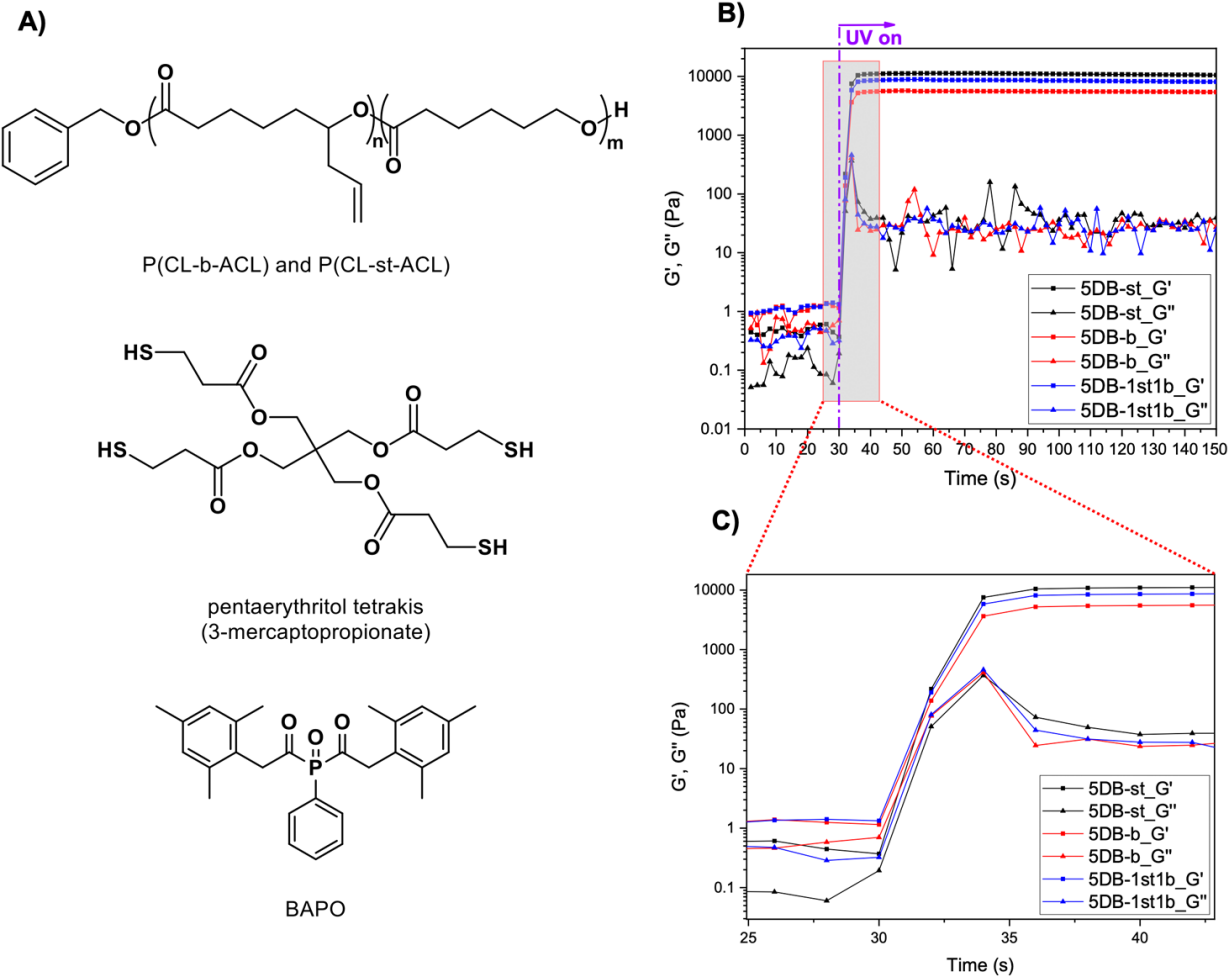
(A) UV-curable resin formulation composition. (B) Photorheology plots; gray box represents the enlarged section (C) with gel points indicated in the dotted box. Polymer concentration is 23.5 wt % in dioxane, thiol cross-linker 1.5 wt %, BAPO 1 wt % (relative to formulation).

Photocurable resins were prepared from the block and statistical copolymers as well as their blends with pentaerythritol tetrakis(3-mercaptopropionate) as tetra-thiol cross-linker. Phenylbis(2,4,6-trimethylbenzoyl)-phosphine oxide (BAPO) (1 wt %; relative to total mass of formulation) was selected as photoinitiator due to its excellent performance under 405 nm UV light and used for the photo-cross-linking of the resins (Figure 2A). 1,4-Dioxane was chosen as a nonreactive diluent due to its high boiling point (101 °C) and miscibility with the caprolactone copolymers. The total concentration of copolymers and cross-linker was kept constant at 25 wt % for all experiments. While all photocurable resins contain the same block and statistical copolymer, their ratio (for the blends) and the ratio of copolymer to tetrafunctional thiol cross-linker were varied, expressed as double bond (DB), obtained from ^1^H NMR analysis, to thiol group (SH) ratio. In this study, resins are identified as **x**DB-**b** or **x**DB-**st**, where **x** stands for the molar ratio of [DB]:[SH], while **b** and **st** refer to the block and the statistical copolymer. For example, 5DB-st refers to a resin from the statistical copolymer with a molar ratio of [DB]:[SH] = 5. Resins from copolymer blends are identified as **x**DB-**y**st**z**b, where **y** and **z** refer to the mass ratio of statistical to block copolymer; for example 5DB-1st1b refers to a 1:1 mixture of both copolymers with a [DB]:[SH] = 5.

Photorheology experiments were first carried out on three resins to investigate their photoreactivities. While we clearly observed the separation between the storage modulus (*G*′) and the loss modulus (*G*″), indicating the gelation process, we were unable to precisely identify the gel point, which is typically characterized by the crossover of *G*′ and *G*″. We hypothesized that the liquid resins exhibit viscoelastic behavior, where the storage modulus is higher than the loss modulus prior to irradiation, making the identification of the gel point not possible (Figure 2C). After 6 s the storage modulus reached a plateau, which indicates that the ultimate degree of cross-linking was reached (Figure 2C). These results confirm that the three resin formulations exhibit excellent photoreactivity with rapid cross-linking, thus making them ideal candidates for use in 3D DLP printing.

The solvent-induced swelling degree of various PCL copolymer networks was examined in 1,4-dioxane (Figure S3) on dried cross-linked films. Ten different formulations with varied thiol/ene ratios as well as polymer compositions were examined, as summarized in Table 1. Generally, with a decreasing [DB]:[SH] ratio a higher gel fraction was observed, reaching a maximum of ca. 95%. This is associated with a lower mass swelling ratio, as expected due to the higher cross-link density. When comparing the networks obtained from block and statistical copolymers with the same [DB]:[SH] ratio, for example, 5DB-st and 5DB-b, the block copolymer displays a higher swelling ratio and a lower gel fraction. This suggests a higher cross-link density for the statistical copolymer most likely due to better accessibility of the allyl groups as compared to the sterically more demanding double-bond configuration in the block copolymer. The samples from the copolymer blends show a similar behavior whereby the sample with the highest portion of statistical copolymer (5DB-3st1b) has the lowest swelling ratio and the highest gel fraction for the same [DB]:[SH] ratio. This provided first evidence that the properties of the materials can be engineered by the structure of the copolymer.

**Table 1:** Swelling Study of Polymer Films Obtained from Photocuring in a Mold

Resin^a^	Molar ratio [DB]:[SH]	Swelling ratio^b^	Gel fraction^b^ %
2DB-st	12.5:1	9.7 ± 0.4	85.3 ± 1.0
5DB-st	5:1	5.2 ± 0.1	94.9 ± 0.4
7DB-st	3.6:1	4.4 ± 0.1	95.3 ± 1.0
10DB-st	2.5:1	3.8 ± 0.1	94.3 ± 0.3
2DB-b	12.5:1	12.2 ± 0.3	76.6 ± 0.2
5DB-b	5:1	6.8 ± 0.2	88.4 ± 0.0
7DB-b	3.6:1	6.4 ± 0.5	87.2 ± 0.7
10DB-b	2.5:1	4.1 ± 0.1	93.5 ± 0.3
5DB-3st1b	5:1	5.0 ± 0.1	93.1 ± 0.7
5DB-1st1b	5:1	5.8 ± 0.2	91.7 ± 1.9
5DB-1st3b	5:1	6.7 ± 0.2	88.2 ± 1.1

a Copolymer plus thiol cross-linker concentration 25 wt % in dioxane, BAPO 1 wt %.

b Standard deviation of *n* = 3.

**Figure 3:**
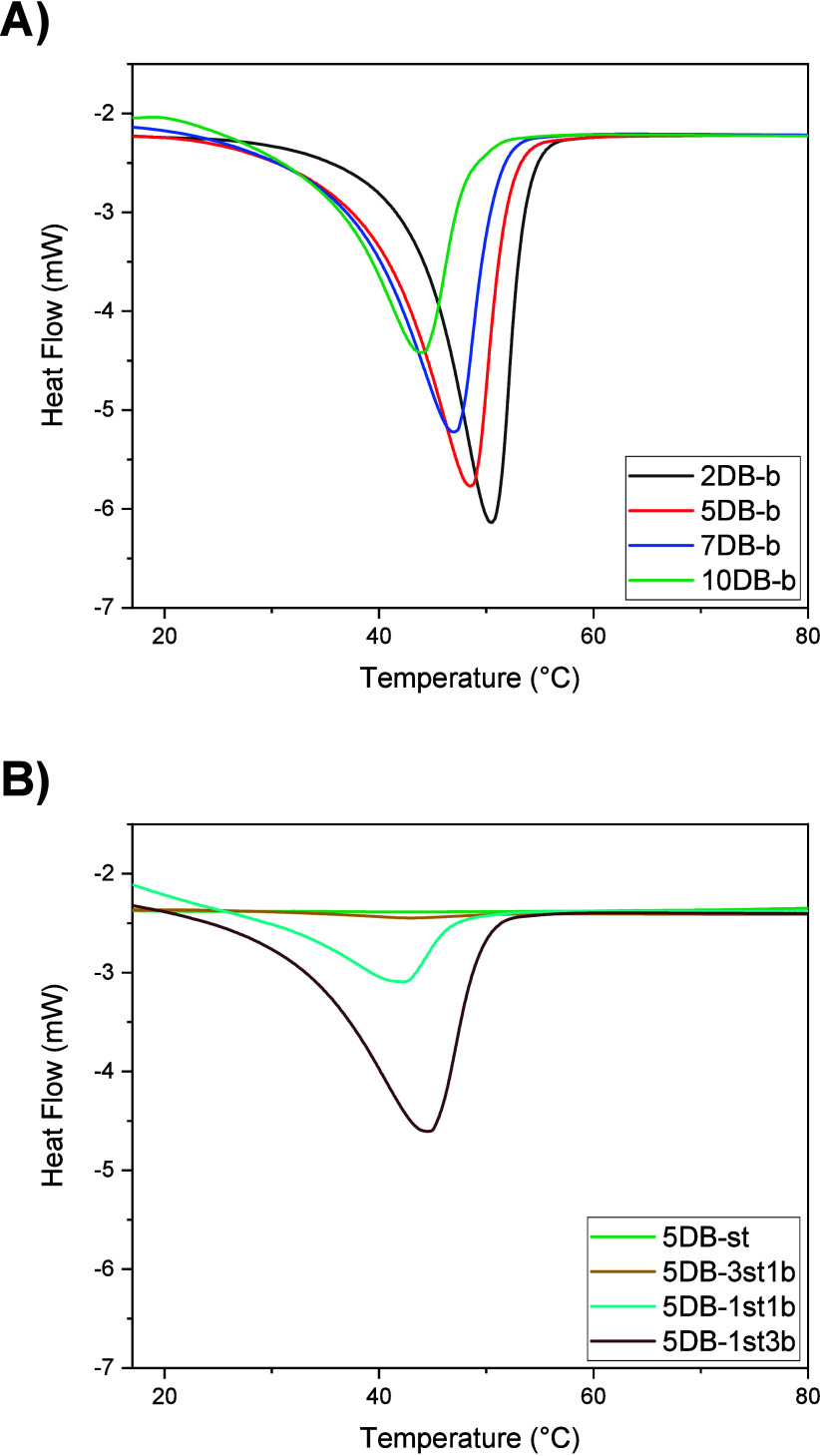
DSC thermograms of networks obtained from block copolymers at different [DB]:[SH] ratios (A) and statistical as well as copolymer blend networks (second heating cycles) (B). Copolymer plus thiol cross-linker concentration 25 wt % in dioxane, BAPO 1 wt %.

DSC was used to study the thermal properties of the dry cross-linked networks. Thermograms in Figure 3A show a melting transition for all block copolymer networks due to the crystallinity originating from the PCL block. A clear relationship between the melting enthalpy and the ratio of double bonds/thiols can be observed, in that the melting enthalpy decreases with decreasing [DB]:[SH] ratio, signifying a decrease in crystallinity due to the higher cross-link density (Table 1). For example, as the ratio of [DB]:[SH] increases from 10DB-b to 2DB-b, the melting enthalpy increased from 22.13 J g^–1^ to 35.57 J g^–1^. While crystallization of the PCL block occurs in all networks, a lower cross-link density facilitates crystallization through higher PCL chain mobility as one would intuitively expect. No melting transition was observed for the networks from the statistical copolymer 5DB-st, which is completely amorphous (Figure 3B). Networks from copolymer blends display a melting enthalpy, which scales with an increasing amount of block copolymer for the same [DB]:[SH] ratio (Figure 3B, Table S1). This provides evidence that network properties, here thermal properties, can readily be engineered from a set of two compositionally identical copolymers.

**Figure 4:**
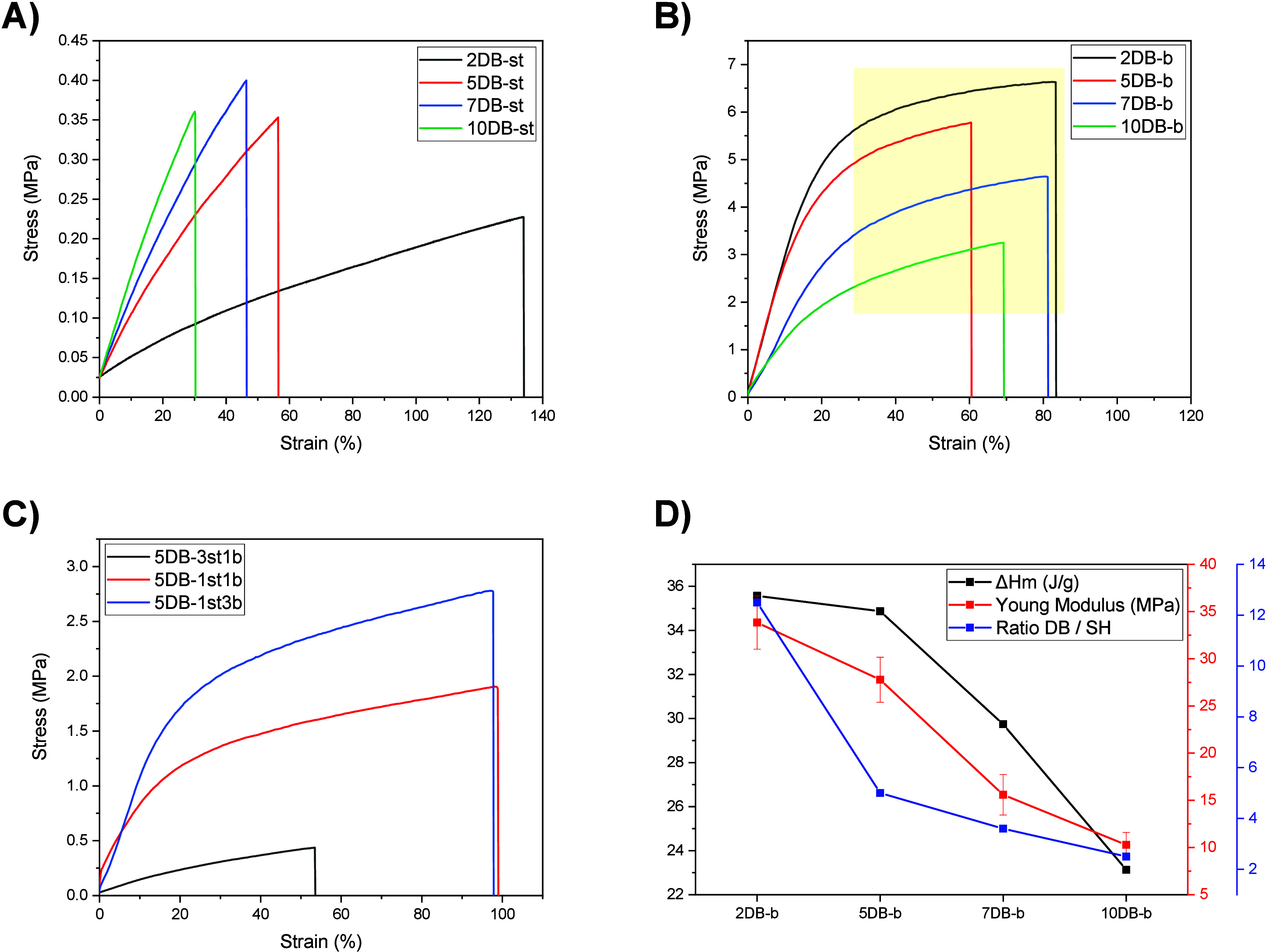
Stress–strain plot of (A) the statistical copolymer networks, (B) the block copolymer networks (the yellow section marks the plastic regime), and (C) copolymer blends (preload of 0.1 N applied). (D) Relationship between melting enthalpy, Young’s modulus, and [DB]:[SH] of the block copolymer networks. Copolymer plus thiol cross-linker concentration 25 wt % in dioxane, BAPO 1 wt %.

To understand the role of crystallization on the mechanical properties, we conducted a series of tensile tests on 10 different networks to determine the Young’s modulus, elongation at break, and ultimate strength (Table 2). It was noticed that for the networks of the amorphous statistical copolymer, the modulus and the ultimate strength increased with the cross-link density, while the elongation at break decreased. This indicates a direct relationship between the modulus and the number of covalent cross-links, i.e., the [DB]:[SH] ratio. An increased cross-link density imparts rigidity to the network, thereby reducing the elasticity of the material. The strain–stress curves for these networks (Figure 4A) show an elastic deformation regime without an observable plastic one, which indicates a limited ability to absorb energy before mechanical failure. Notably, the strain–stress properties of the network from these statistical copolymers meet the requirements of specific soft tissue engineering, which is less achievable with PCL homopolymers.^37^ On the contrary, the networks from the crystalline block copolymer show decreased moduli with increased cross-link density (decreasing [DB]:[SH] ratio). Here the crystalline lattice in the network is the main contributor to the modulus. As discussed above, for crystalline networks, an increased cross-link density leads to a lower melting enthalpy, indicating lower degrees of crystallization, which contributes to the mechanical properties of the network (Figure 4D). Moreover, the stress–strain curves of the crystalline networks exhibit a classic turn from elastic deformation to a plastic deformation region, where crystal plasticity starts dominating the mechanical properties at the yield point. A similar behavior can be observed for the networks from blended statistical and block copolymers, as the crystallinity is contributed by the block copolymer (Figure 4C).

**Table 2:** Mechanical Characterization of the PCL Copolymer Networks^a^

Resin	Molar ratio [DB]:[SH]	Young’s modulus (MPa)	Elongation at break (%)	Ultimate strength (MPa)
2DB-st	12.5	0.30 ± 0.02	131.66 ± 11.44	0.22 ± 0.12
5DB-st	5	0.89 ± 0.03	55.40 ± 18.06	0.33 ± 0.08
7DB-st	3.6	1.04 ± 0.04	45.38 ± 8.34	0.40 ± 0.05
10DB-st	2.5	1.26 ± 0.04	30.82 ± 4.53	0.36 ± 0.04
2DB-b	12.5	33.84 ± 2.83	89.86 ± 7.87	5.98 ± 0.50
5DB-b	5	27.7 ± 2.38	59.84 ± 13.51	5.20 ± 0.55
7DB-b	3.6	15.59 ± 2.14	87.38 ± 15.92	4.61 ± 0.34
10DB-b	2.5	10.28 ± 1.31	73.28 ± 5.41	3.36 ± 0.15
5DB-3st1b	5	1.21 ± 0.15	55.16 ± 5.21	0.45 ± 0.04
5DB-1st1b	5	6.69 ± 0.59	101.86 ± 18.05	1.90 ± 0.20
5DB-1st3b	5	11.16 ± 2.12	95.88 ± 13.98	2.78 ± 0.18

a Copolymer plus thiol cross-linker concentration 25 wt % in dioxane, BAPO 1 wt %. Standard deviation of *n* = 3.

Considering that the two copolymers investigated here have an identical composition and only differ in the monomer arrangement (statistical vs block), the mechanical properties of the networks vary significantly. Specifically, comparing the resins 5DB-b and 5DB-st, the modulus of the network of the block copolymer network is 31 times higher than that of the statistical copolymer (27.7 vs 0.89 MPa). Interestingly, intermediate moduli can be obtained by blending the two copolymers. For example, the network of resin 5DB-1st1b, formulated from 50% block and 50% statistical copolymer, displays a modulus of 6.69 MPa. These differences underline the role of crystallinity in the material’s mechanical characteristics. The presence of crystalline blocks in the block copolymer imparts a higher degree of organization, which can be tuned by the cross-link density, resulting in enhanced mechanical strength and stiffness. Intriguingly, this diverse range of mechanical properties can be engineered into the cross-linked network through copolymer architecture and cross-link density without changing the overall copolymer composition.

### DLP 3D-Printing of Thiol–Ene Cross-Linkable PCL Copolymers

**Figure 5:**
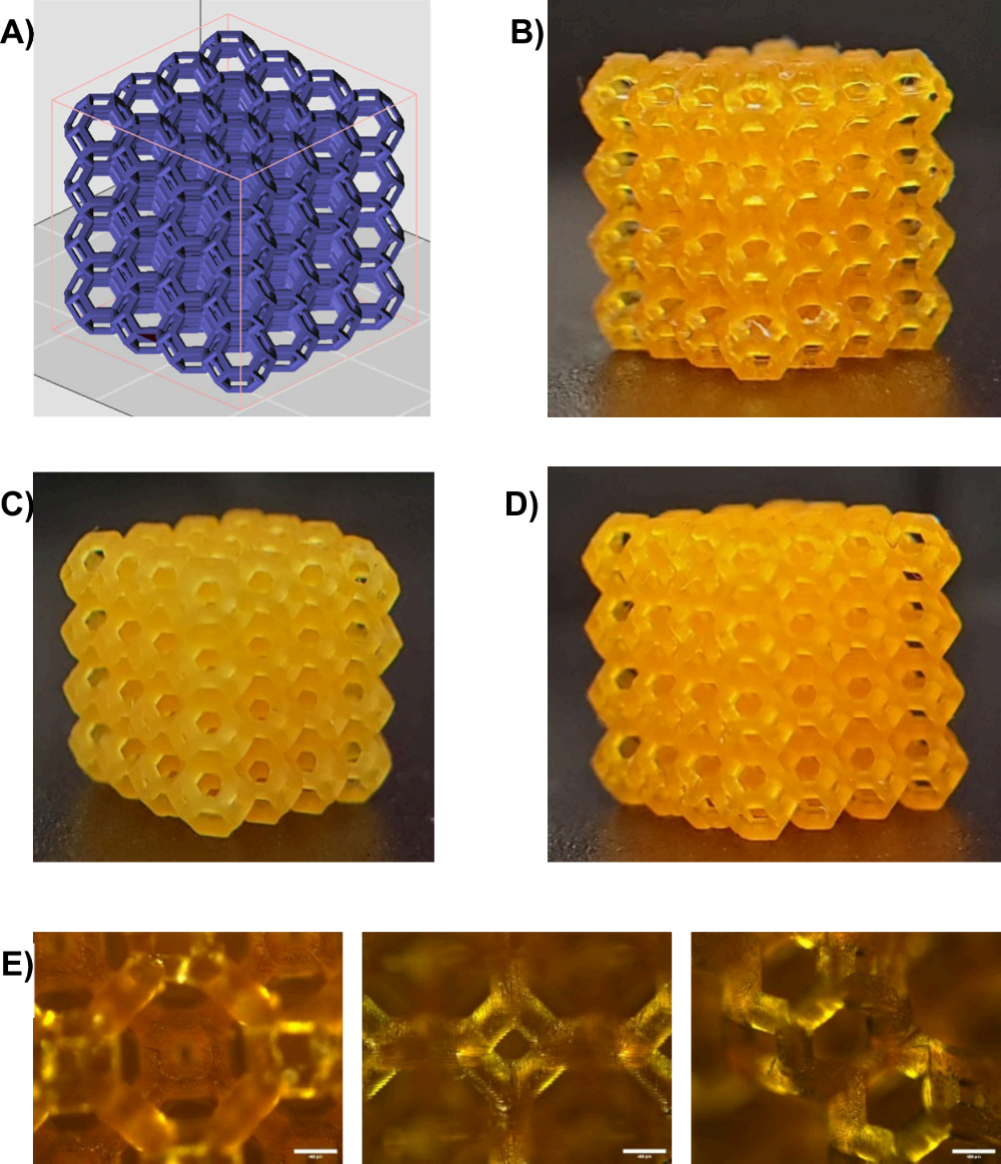
(A) 3D digital model. (B) 3D-printed scaffold of 5DB-st. (C) 3D-printed scaffold of 5DB-b. (D) 3D-printed scaffold of 5DB-st. (E) Zoom-in image of 3D-printed scaffold (5DB-st) from different surfaces; scale bar 400 μm. All the resins were printed in the same design dimensions (6 mm × 6 mm × 6 mm). Printing conditions: Copolymer plus thiol cross-linker concentration 25 wt % in dioxane, BAPO 1 wt %, Sudan I 0.05 wt %, pyrogallol 0.5 wt %, light intensity 22 mW cm^–2^, layer exposure time of 3 s per layer (height 50 μm). After printing, objects were washed with acetone and postcured for 10 min under UV light (405 nm, 6 mW cm^–2^). Objects were dried in a vacuum oven at 40 °C overnight.

We utilized the statistical copolymer 5DB-st, the block copolymer 5DB-b, and the blend of block and statistical copolymers 5DB-1st1b for DLP printing. 1,4-Dioxane was chosen as a solvent for its high boiling point, preventing evaporation during printing. The viscosities of all three resins at 25 wt % were measured by rheology to facilitate efficient utilization in DLP (32.1, 31.9, and 27.9 mPa·s, respectively, Figures S4–S6). To enable printing, a photoabsorber (Sudan I, 0.05 wt %) was added to optimize the Z axis resolution, allowing control over the light penetration during the printing process. Additionally, pyrogallol (0.5 wt %), a radical inhibitor, was added to prevent free radical polymerization, without affecting the photoreactivity of the resin. A porous lattice cube was chosen as a 3D model (Figure 5A), due to its complexity allowing to challenge the efficiency of the resins and the printing process. The structure was successfully printed using a light intensity of 22 mW/cm^2^, with a layer exposure time of 3 s per layer (height 50 μm). Before imaging, all solvent was removed from the structures. The high fidelity of the 3D-printed lattices to the original design underscores the ability of the resin to achieve fine-scale resolutions and intricate geometries (Figure 5). The 3D objects present an elevated level of accuracy, capable of reproducing small size pores up to ∼400 μm (Figure 5D).

**Figure 6:**
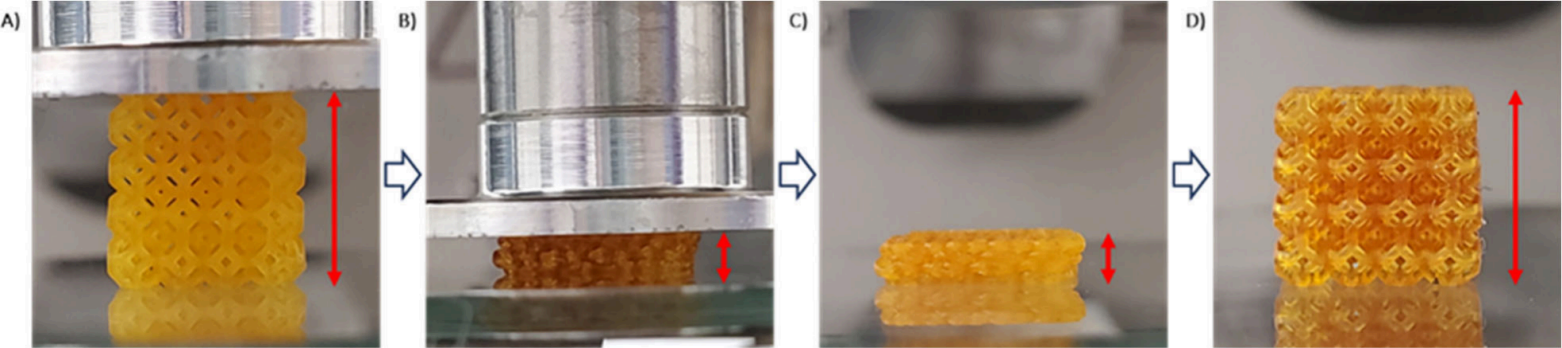
Shape memory effect for the 3D-printed structure from the resin 2DB-b. (A) Original 3D-printed structure. (B) Deformed structure under heating. (C) Fixed deformed shape after cooling. (D) Original shape after reheating the structure.

A significant advantage of manufacturing semicrystalline polymers is the display of a thermal-responsive shape memory property. Shape memory materials are able to recover to the original shapes upon stimulations and are attractive for many applications.^38−40^ A common strategy to fabricate a shape memory thermoset in 3D printing is assisted by a transition temperature including melting temperature (*T*_*m*_) and glass transition temperature (*T*_*g*_).^41^ Upon heating, the structure of a semicrystalline thermoset is temporarily deformable by melted segments, whereas it can recover to its printed permanent shape undergoing another thermal transition process.^42^ Shape memory properties of a DLP-printed PCL thermoset have rarely been reported.^43^ Encouraged by the thermal properties of the crystalline polymer networks, we examined the shape memory of the 3D-printed lattice by exploiting the reversible melting and recrystallization processes. The shape memory behavior was studied for the lattice printed from the crystalline block copolymer containing resin 2DB-b. A sequence of steps was followed to induce the shape memory phenomena (Figure 6). First, the as-printed 3D structure was heated above the melting point of its network (50.46 °C), where it exhibits a more deformable state. Then, by employing an external force, the object was compressed to 25% of its original height. Before removing the external force, the structure was cooled to room temperature to allow crystallization, causing the structure to retain its deformed shape. Eventually, the compressed thermoset was able to rapidly recover to its original shape by heating above its *T*_*m*_, where the PCL block regains mobility (video in the SI). The same experiment was carried out with 2DB-st containing the amorphous copolymer, but no structural stabilization was observed upon deformation. This proves the one-way shape memory of the block copolymer scaffold is attributed to the crystallinity of the PCL block.

### Degradation and Cell Compatibility

**Figure 7:**
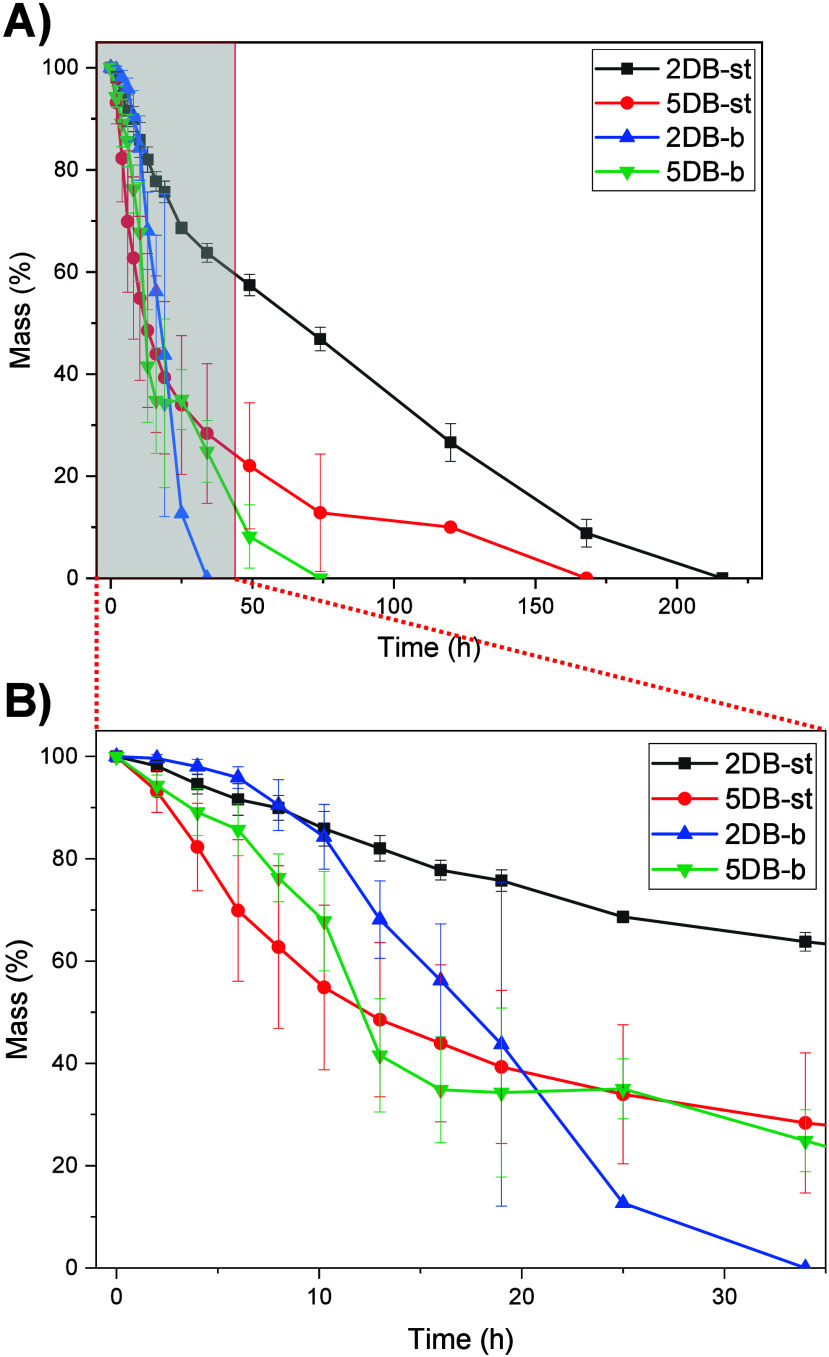
Degradation test in 5 M NaOH at room temperature. (B) Zoom-in of the red region of the (A) graph. Error bars represent standard deviation (*n* = 3).

Accelerated hydrolytic degradation tests were conducted on four copolymer networks (Table S2) to demonstrate the effect of crystallinity and cross-link density on degradation. The tests were performed by immerging cross-linked resin discs in 5 M NaOH and evaluating their mass loss over time at room temperature (Figure 7). Degradation commonly proceeds through surface erosion of the amorphous regions and erosion and fragmentation of the crystalline regions.^44^ The amorphous discs remained structurally intact until they approached near-complete mass loss, leaving the solution clear, indicating complete degradation and dissolution (Figure S8). Notably, amorphous PCL networks with higher cross-link density (2DB-st vs 5DB-st) were observed to have a faster degradation. This might suggest that ester bonds from a polymer backbone with adjacent thiol–ether linkage are more prone to hydrolysis.^45^ The degradation rate of the semicrystalline discs was similar to the amorphous ones for the first 8 h of degradation. As the amorphous region in the semicrystalline network experienced statistical hydrolysis, the highly ordered crystalline polymer chain can be deconstructed from the network. The weight changes of disks therefore are consistent with fragmentation and surface erosion after 8 h. The overall results suggested the possibility to optimize the degradation of the thiol–ene PCL network from its crystallinity and cross-link density.

Finally, the cytotoxicity of printed statistical and block copolymer scaffolds (5DB-st, 5DB-b) were investigated in the Calu-3 cancer cell line. The viability of the cells was determined compared to untreated cells. Overall, both scaffolds exhibit cell viability over 100% relative to the untreated control over the tested period (7 days) (Figure S9), confirming their cytocompatibility in keeping with the known cell compatibility of PCL.

## Conclusions

This study introduced copolymers of caprolactone and allyl-caprolactone with inherent photoreactive functionalities that can be exploited in DLP 3D printing technology by thiol–ene cross-linking. Depending on whether the resin comprises a copolymer with a block or statistical chain structure, markedly different thermal, mechanical, and degradation properties were obtained for otherwise identical copolymer compositions. Through the blending of statistical and block copolymers, it is possible to realize material properties in between those of the base resins. This enables the exact engineering of material properties of cross-linked polymer networks to match the desired application. These resins display fast photocuring kinetics and enable precision DLP printing of high-resolution objects, where the different properties of the materials are retained as demonstrated for the thermal shape-memory materials. We believe this could potentially be impactful given the importance of PCL as a biocompatible polymer used in a multitude of medical devices and biomaterials. Using only two base copolymers, it would allow bioengineers to dial in the required mechanical and degradation properties to match the cell types and side of action requirements, while having the flexibility to process the materials to the desired 2D or 3D shape.

## Experimental Section

### Materials

All chemicals were used as received, unless stated otherwise. 3-Chloroperoxybenzoic acid, NaHCO_3_, CH_2_Cl_2_, Celite 281, methylene chloride, NaCl, pyrogallol, NaOH, tetrakis(3-mercaptopropionate) pentaerythritol, 1,4-dioxane, phenylbis(2,4,6-trimethylbenzoyl)phosphine oxide (BAPO), tin(II) octoate, and benzyl alcohol were purchased from Sigma-Aldrich. 2-Allyl cyclohexanone and ε-caprolactone were purchased from Tokyo Chemical Industry. Sudan I and MgSO_4_ were purchased from Fluorochem.

### Copolymer Synthesis

The statistical copolymer of 6-allyl-ε-caprolactone and ε-caprolactone was synthesized by ring-opening polymerization of 5.707 g (50 mmol) of ε-caprolactone and 3.85 g (25 mmol) of 6-allyl-ε-caprolactone using benzyl alcohol 108 mg (1 mmol) as initiator. Tin(II) octoate (40 mg, 0.167 mmol) was used as catalyst, and the reaction proceeded in bulk at 110 °C for 48 h under nitrogen. The reaction was followed by ^1^H NMR until monomers conversion >99% was reached. The block copolymer was synthesized by a two-step polymerization. Initially, the poly allyl-ε-caprolactone block was synthesized by mixing 3.85 g (25 mmol) of allyl-ε-caprolactone and 108 mg (1 mmol) of benzyl alcohol and 40 mg (0.167 mmol) of Tin(II) octoate in a Schlenk flask. The flask was then purged with N_2_ and heated at 110 °C overnight. The consumption of allyl-ε-caprolactone (99%) was confirmed by ^1^H NMR. A 5.707 g (50 mmol) amount of ε-caprolactone was added into the reaction under a N_2_ flow and left to react for 5 days. ^1^H NMR analysis confirmed a generally slower initiation from the macroinitiator as well as slower reaction kinetics after 70% of monomer conversion. The final product was characterized by ^1^H NMR, DOSY NMR, and SEC. ^1^H NMR (400 MHz, CDCl_3_, 299 K, ppm): δ = 7.40–7.30 (m, 5H, Ar), 5.72 (ddt, 25H, C*H*=CH_2_), 5.18–5.01 (m, 50H, CH=C*H*_2_), 5.06 (s, 2H, C=OOC*H*_2_Ar), 4.05 (t, 100H, C*H*_2_OC=O), 2.25 (t, 150H, C*H*_2_C=OO), 1,73–1.14 (m, C*H*_2_CH_2_C*H*_2_, CH_2_C*H*_2_CH_2_) ppm.

### Characterization Methods

#### Nuclear Magnetic Resonance (NMR)

^1^H and ^1^H diffusion ordered spectroscopy (DOSY) spectra were recorded using a Bruker Avance 400 MHz spectrometer at room temperature. All chemical shifts were reported in parts per million (ppm) relative to the chloroform reference peak at δ = 7.26 ppm, while diffusion coefficients are reported in cm^2^ s^–1^.

#### Gel Permeation Chromatography (GPC)

Molecular weight distributions and polydispersity indexes were determined by a CHCl_3_ Agilent Technologies LC 1200 Series equipped with an Agilent 1260 ISO pump, Agilent refractive index detector, and two columns. Samples were dissolved in CHCl_3_, and their chromatograms recorded with a flow of 1.0 mL/min at 40 °C. The system was calibrated against PSS Polymer Standards Service GmbH linear poly(methyl methacrylate). All GPC samples were prepared at a concentration of 2 mg/mL and were filtered through a 0.2 μm Millipore before injection.

#### Differential Scanning Calorimetry

DSC measurements were performed using a TA Instruments DSC Q200 and TA Instruments RSC FC-100 immersion cooler, with 5–7 mg of the dry cross-linked material as a sample. A heating and cooling rate of 10 °C per minute was used. The second heating and cooling cycles were used to analyze the thermal properties. Each sample was measured in an aluminum Tzero pan under nitrogen flow using an empty pan as reference.

#### Resin Viscosity

The viscosity was measured using an Anton Paar modular compact rheometer (MCR) 301 using a Peltier hood to protect the sample from ambient light. The test chosen is used to measure the viscosity of quasi-Newtonian liquids. The experiment was conducted at room temperature with a shear rate range of 1 to 100 s^–1^, collecting 100 data points over 550 s. A regression curve was calculated representing the best fit of the data to a quasi-Newtonian rheological model.

#### Photorheology

Photorheology experiments were carried out using an Anton Paar MCR 301. The machine was equipped with a Thorlabs UV LED light 405 nm (M405L3-C1) and a sample glass plate allowing the passage of light. The experiments were conducted at room temperature using a Peltier hood to protect the sample from ambient light. A parallel plate of 25 mm diameter was used with a gap length of 0.05 mm. Each time point was taken every 10 s through a time sweep experiment with constant oscillations at a fixed frequency of 10 rad/s with a strain of 0.1%. UV light (6 mW/cm^2^) was turned on after 30 s.

### Sample Preparation for DSC, Swelling Tests, and Tensile Tests

The resins were poured into rectangular molds (H0.8 × W10 × L20 mm) and irradiated with UV light of 405 nm (2 mW cm^–2^) for 1 h at room temperature. Afterward, the cross-linked sheets were washed with acetone and postcured for 10 min under UV light (405 nm, 6 mW cm^–2^) followed by drying in a vacuum oven (40 °C) overnight. After drying the size of the sheets became H0.4–0.5 mm × W7–8 mm × L14–16 mm. The samples prepared in this way were used to conduct DSC, the swelling test, and the tensile test.

### Swelling Test

Gel fraction and swelling ratio were calculated on cross-linked sheets prepared as described. The cross-linked sheets were dried in a vacuum oven (40 °C) overnight to ensure the removal of the diluent, then cooled down at room temperature and weighted (initial dry mass *W*_*d*_). The discs were immersed in excess 1,4-dioxane for 48 h at room temperature, then weighted to obtain the swollen mass (*W*_*s*_). Next, the swollen discs were dried in a vacuum oven (40 °C) overnight to then measure the dry mass after swelling (*W*_*a*_). Using the following equations, the gel fraction and swelling ratio were calculated. All the measurements were performed in triplicate.
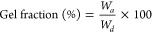

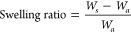


### Tensile Test

Tensile testing was carried out using a Testometric M100-1CT machine equipped with a 50 N cell load (LC5). Rectangular cross-linked sheets were used to perform the measurements, obtained according to the procedure described above. A gauge length of 8 mm, pretension of 0.1 N, and test speed of 10 mm min^–1^ were used as parameters for the machine. The tests were performed at room temperature. Young’s modulus, elongation at break, and ultimate strength have been determined as averages of five independent drawing experiments performed at the same conditions.

### 3D Printing

3D printing was performed using a custom DLP 3D printer, MONO3-2K40 (Monoprinter). The projector resolution was 1902 × 1080 pixels, with an in-plane resolution of 15 μm. Each layer thickness was 50 μm, with an exposure time of 8 s for the first two base layers and 3 s for all subsequent layers. A light intensity of 22 mW cm^–2^ (measured on the surface of the tank) was used for all the printings at room temperature. The porous lattice cube 3D model was downloaded from Thingverse (https://www.thingiverse.com/thing:2522147) designed by ProFab3D under Creative Commons – Attribution license. The resin composition includes 23.5 wt % P(CL50-ACL25) (block and/or random), 1.5 wt % pentaerythritol tetrakis(3-mercaptopropionate), 1 wt % BAPO, 0.05% wt Sudan I, 0.5% wt pyrogallol, and 74% wt 1,4-dioxane. After printing, objects were washed with acetone and postcured for 10 min under UV light (405 nm, 6 mW cm^–2^). Objects were dried in a vacuum oven at 40 °C overnight.

### Shape Memory Effect Procedure

Shape memory studies were done using an Anton Paar 301 MCR. The 3D-printed structure was heated at 60 °C for 5 min using the integrated heated glass plate of the rheometer. Then, the external force was applied via the rheometer shaft equipped with a parallel plate for 30 min to allow the structure to cool to room temperature. Finally, after the removal of the external force, the structure was heated to 60 °C using the rheometer plate for 5 min.

### Accelerated Degradation

A solution of 5 M NaOH was used to perform the degradation test. Sample were prepared as described before but using a disc-shaped mold. After the removal of the diluent discs of H1.3 × Ø5 mm were obtained. Each sample was immersed in 3 mL of NaOH solution. The discs were removed at each time point, washed with deionized water, dried with paper, and weighed together before being added back to the solution. The test was carried out at room temperature in triplicate for each resin analyzed.

### Cytotoxicity

The cytotoxicity of the caprolactone scaffold on the metabolic activity of calu-3 cells post-co-seeding was determined by the AlamarBlue assay (ThermoScientific, Ireland). AlamarBlue is a non-end point assay (nondestructive assay). Cells were maintained and seeded in DMEM/F12 ham supplemented with 10% fetal bovine serum and 1% penicillin/streptomycin mixture solution (complete media). The cells were seeded at 50 × 103 cells/well in a 12-well culture plate and allowed to attach overnight at 37 °C/5% CO_2_. The cells were treated for further incubation in the presence of a small specimen of gel (∼0.5 cm^3^). On each specific day, the cells were treated for 2 h in the presence of 10% AlamarBlue in complete media, and the assay was carried out for up to 7 days. Subsequently aliquots of resultant supernatant were transferred to a 96 black well plate and the fluorescence was determined using a Tecan Pro100 plate reader at an excitation wavelength of 545 nm with an emission wavelength of 570 nm. The relative calu-3 metabolic activity was expressed as a percentile of treated to untreated cells.

## Supporting Information Available

Synthesis and characterization of allyl-caprolactone, DOSY NMR of copolymers, table of thermal properties of copolymers, table of resins used for degradation, images of cured resins and degradation experiments, cytotoxicity results (PDF)
Video of shape memory experiment (MP4)
