# Supplementary material for: Digital Light Processing (DLP) 3D Printing of Caprolactone Copolymers with Tailored Properties through Crystallinity
Source: ACS Appl Polym Mater. 2024 Sep 16;6(18):11241–50. doi: 10.1021/acsapm.4c01772 (PMC11443484; doi:10.1021/acsapm.4c01772)
Supplement: Supplementary file 1 — ap4c01772_si_001.pdf [file ap4c01772_si_001.pdf]

Supporting Information

Digital Light Processing 3D Printing of Caprolactone Copolymers with Tailored Properties through Crystallinity

Gianluca Bartolini Torres,[1,2] *Smiljana Stefanovic,*[1] *Bo Li,*[1,3]* *Andreas Heise*[1,2,3] *

[1]Department of Chemistry, RCSI University of Medicine and Health Sciences, Dublin, D02 YN77, Ireland. [2]Science Foundation Ireland (SFI) Centre for Research in Medical Devices (CURAM), RCSI, Dublin, D02 YN77, Ireland [3]AMBER, The SFI Advanced Materials and Bioengineering Research Centre, RCSI, Dublin, D02 YN77, Ireland.

Email: andreasheise@rcsi.ie

Synthesis of 6-Allyl-ε-caprolactone: 6-Allyl-ε-caprolactone was synthesized following literature methods.[1] 19.54 g (87.2mmol) of 3-chloroperoxybenzoic acid (77%) was added to a mixture of 2-allyl cyclohexanone 10g (72.4 mmol), $NaHCO_3$ 9 g (108.6 mmol), and 360mL of CH_2Cl_2 at 0 °C. The mixture was stirred at room temperature for 72 hours. The suspension was then cooled to 0˚C, filtered over Celite, and washed with saturated aqueous $NaHCO_3$ solution and with brine. The organic fraction was dried with $MgSO_4$ and concentrated under reduced pressure. The final product was obtained by distillation with vacuum as a light yellow oil (8.2 g, yield = 74%). ^{1}H NMR (400 MHz, $CDCl_3$, 299K, ppm): δ = 5.82 (ddt, 1H, C*H*=CH_2), 5.24–5.00 (m, 2H, CH=CH_2), 4.27 (dt, 1H, (CO)OC*H*), 2.75–2.51 (m, 2H, CH_2(CO)O), 2.46 (ddd, 1H, CHCH_2CH=CH_2), 2.31 (dt, 1H, CHCH'_2CH=CH_2), 2.01–1.80 (m, 3H, CH_2CH_2CH_2), 1.64–1.35 (m, 3H, CH'_2CH'_2CH'_2).

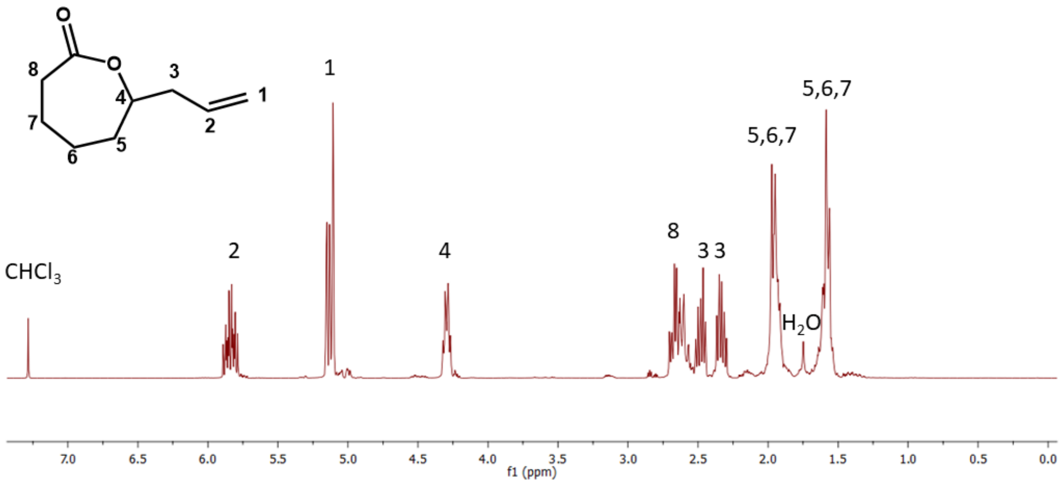

Figure S1. ^{1}H NMR spectra of 6-Allyl-ε-caprolactone in $CDCl_3$.

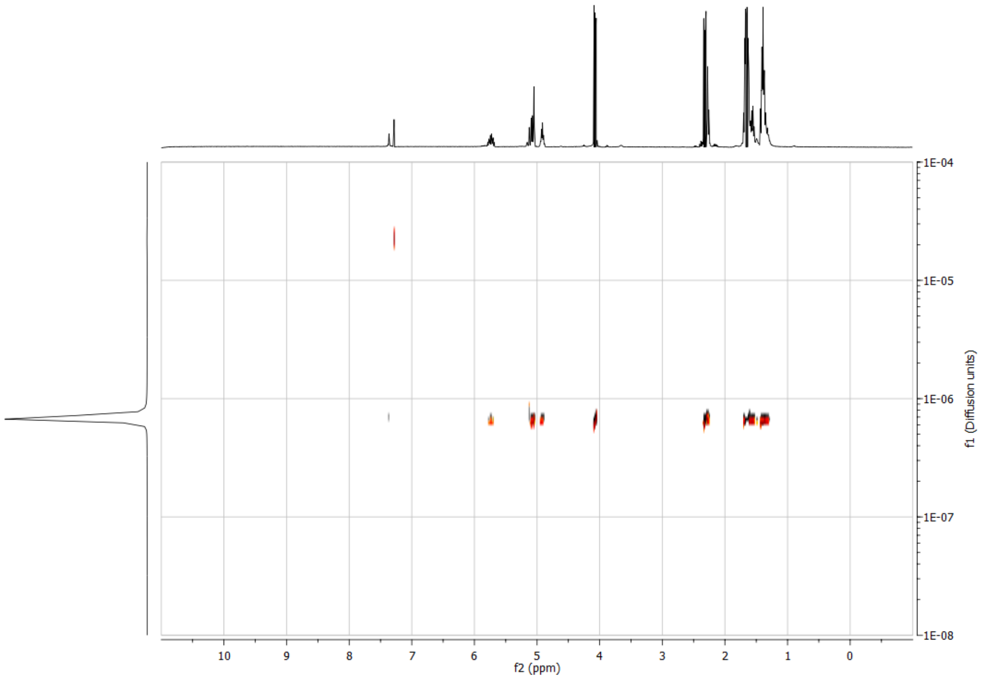

Figure S2. Overlayed DOSY NMR of spectra of P(CL-*st*-ACL) and P(CL-*b*-ACL).

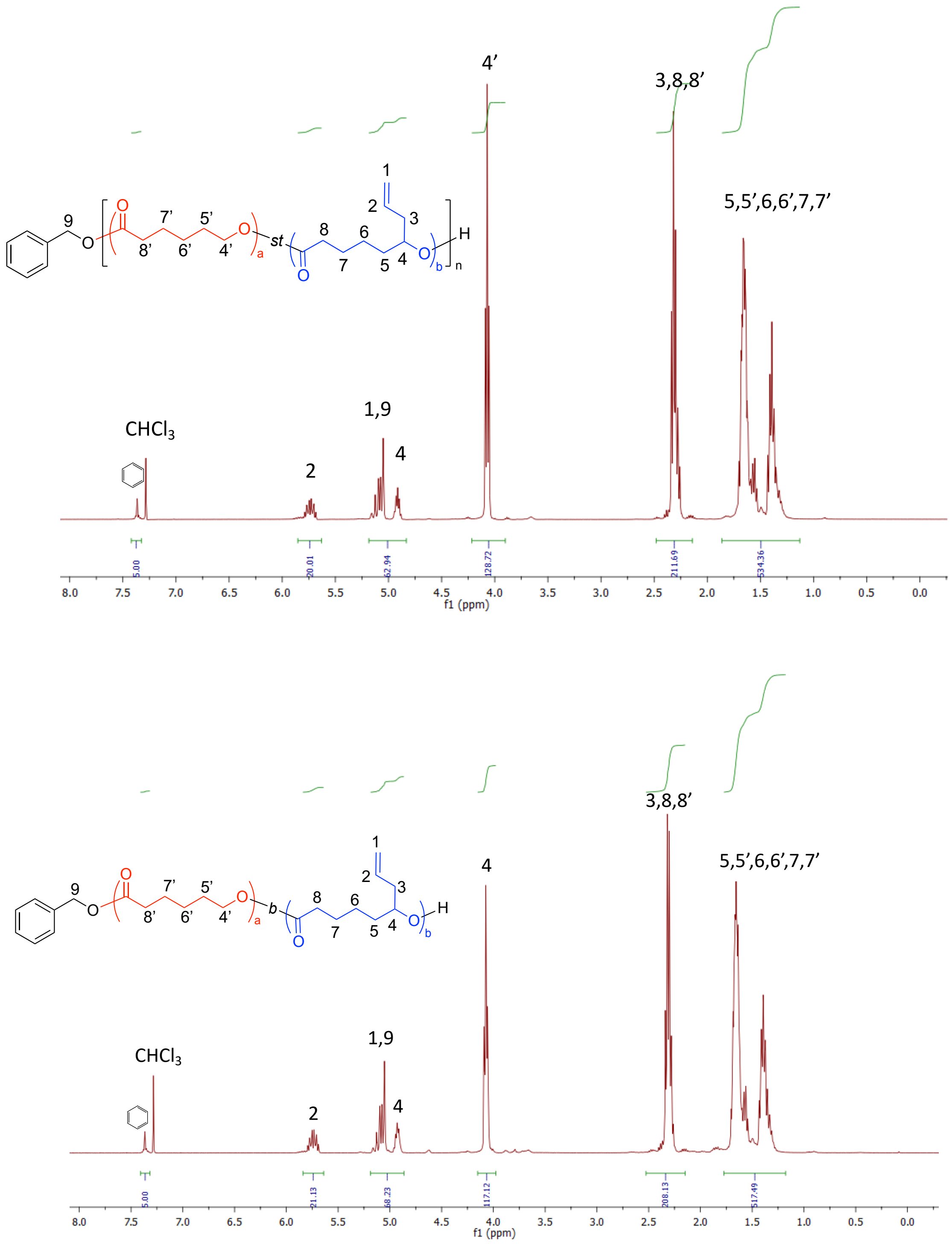

Figure S3. ^{1}H-NMR of spectra of P(CL-*st*-ACL) (top) and P(CL-*b*-ACL) (bottom).

Table S1: Thermal properties of the copolymer networks. Copolymer plus thiol crosslinker concentration 25 %wt in dioxane, BAPO 1 %wt.

Resin	Molar Ratio [DB]:[SH]	T_c (°C)[a]	ΔH_c (J/g)[b]	T_m (°C)[c]	ΔH_m (J/g)[d]
5DB-st	5:1	-	-	-	-
2DB-b	12.5:1	9.6	35.24	50.5	35.57
5DB-b	5:1	2.6	30.87	47.1	34.87
7DB-b	3.6:1	0.2	23.10	46.9	29.74
10DB-b	2.5:1	-1.6	1.48	43.8	22.13
5DB-st1b	5:1	-1.9	0.04	12.6	1.41
5DB-1st1b	5:1	-8.8	0.45	42.2	9.79
5DB-1st3b	5:1	-15.7	3.15	44.5	24.88

[a] = Crystallization temperature. [b] = Crystallization enthalpy. [c] = Melting temperature. [d] = Melting enthalpy.

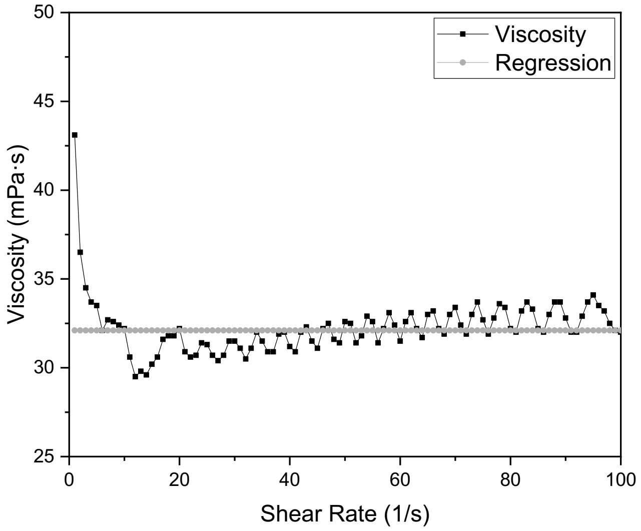

Figure S4. Viscosity results for the resin 5DB-st. Black curve: Viscosity vs Shear Rate. Grey curve: Regression curve represents the best fit of the data to a quasi-Newtonian rheological model (32.1 mPa·s). The horizontal regression line indicated a constant viscosity regardless of the shear rate typical for a Newtonian fluid.

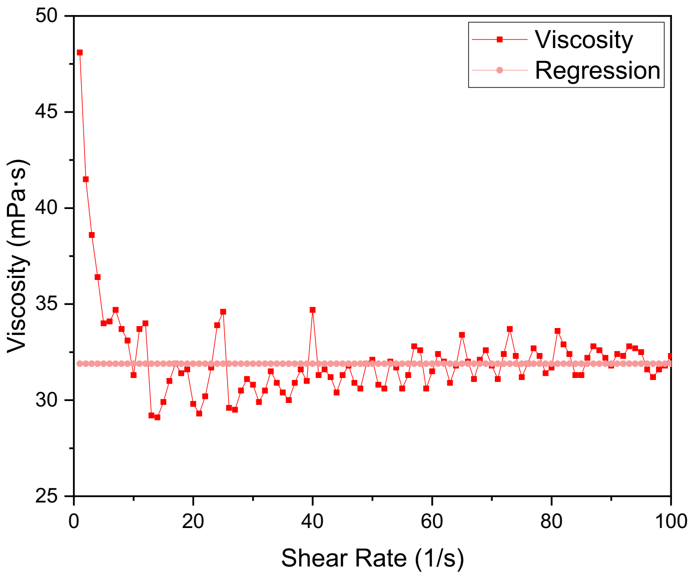

Figure S5. Viscosity results for the resin 5DB-b. Red curve: Viscosity vs Shear Rate. Pink curve: Regression curve represents the best fit of the data to a quasi-Newtonian rheological model (31.9 mPa·s). The horizontal regression line indicated a constant viscosity regardless of the shear rate typical for a Newtonian fluid.

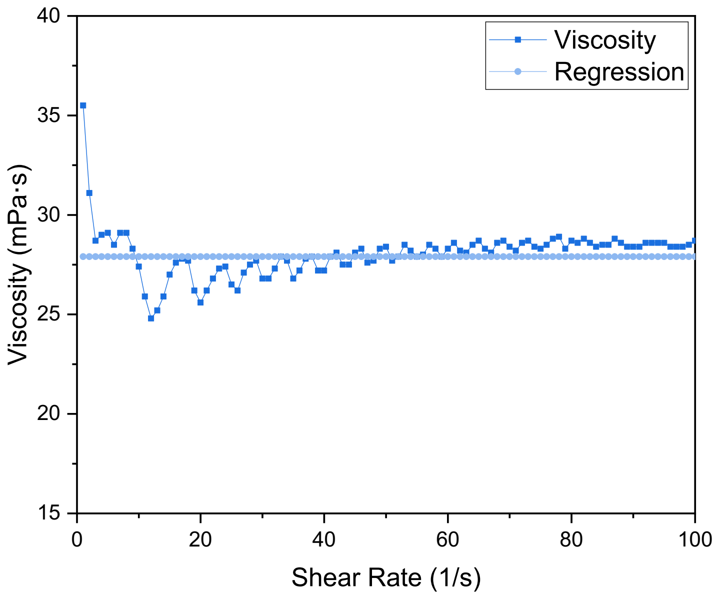

Figure S6. Viscosity results for the resin 5DB-1st1b. Blue curve: Viscosity vs Shear Rate. Light blue curve: Regression curve represents the best fit of the data to a quasi-Newtonian rheological model (27.9 mPa·s). The horizontal regression line indicated a constant viscosity regardless of the shear rate typical for a Newtonian fluid.

Table S2. Crosslinked networks used for the degradation tests.

Resin	*Polymer composition*	*Polymer %wt*	*%wt thiol crosslinker*	*BAPO %wt*	*Molar Ratio [DB]:[SH]*	*ΔHm (J/g)*
**2DB-st**	P(CL$_{50}$-*st*-ACL$_{25}$)	24.4	0.6	1	12.5	-
**5DB-st**	P(CL$_{50}$-*st*-ACL$_{25}$)	23.5	1.5	1	5	-
**2DB-b**	P(CL$_{50}$-*b*-ACL$_{25}$)	24.4	0.6	1	12.5	35.57
**5DB-b**	P(CL$_{50}$-*b*-ACL$_{25}$)	23.5	1.5	1	5	34.87

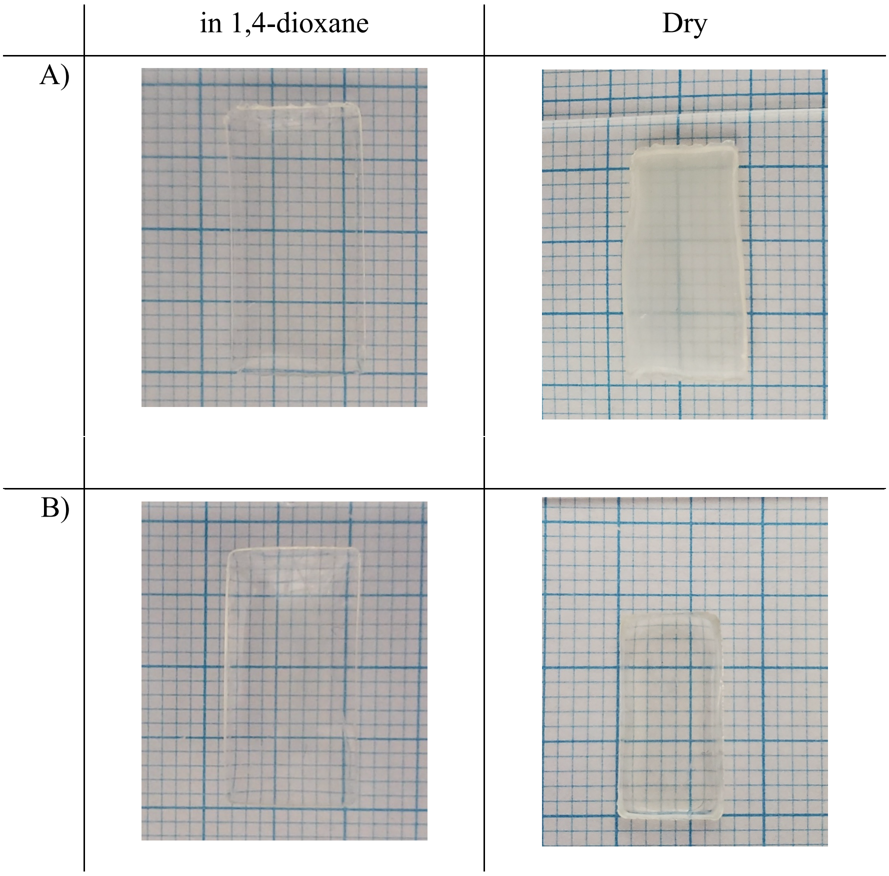

Figure S7. Pictures of (A) 5DB-b and (B) 5DB-st resin crosslinked films.

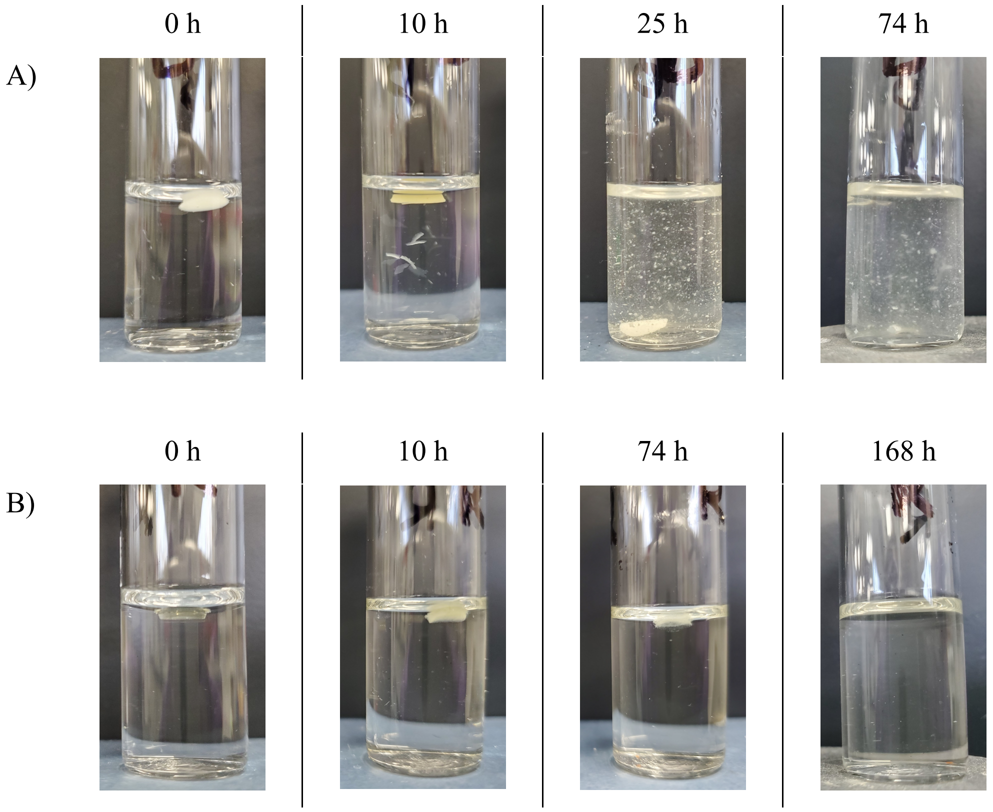

Figure S8. Accelerated degradation of statistical and block crosslinked discs in 5M NaOH at room temperature, for (A) 5DB-b and (B) 5DB-st resins.

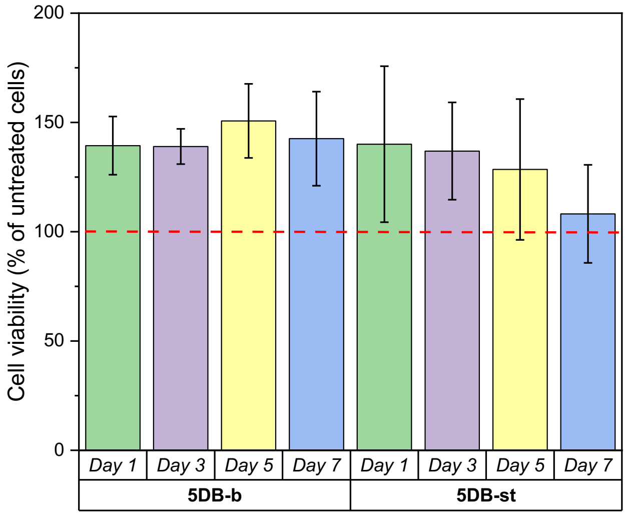

Figure S9. Cytotoxicity of printed statistical and block scaffolds. Metabolic activity was determined by AlamarBlue® assay 7 days post-co-seeding the cells with fractions of 5DB-b and 5DB-st scaffolds. Results were plotted as percentile of metabolic activity relative to untreated cells. Data shown as mean ± SD (Triplicate measurement per well). $p > 0.05$ values, Two-way ANOVA with Dunnett posthoc analysis compared with control (Day 1) for 5DB-st and 5DB-b scaffolds, respectively.

References

Mecerreyes, D.; Miller, R. D.; Hedrick, J. L.; Detrembleur, C.; Jérôme, R. Ring-opening polymerization of 6-hydroxynon-8-enoic acid lactone: Novel biodegradable copolymers containing allyl pendent groups. *J. Polym. Sci. A: Polym. Chem.* **2000**, *38*, 870-875.
